# A pilot study on the effect of d-allulose on postprandial glucose levels in patients with type 2 diabetes mellitus during Ramadan fasting

**DOI:** 10.1186/s13098-022-00856-3

**Published:** 2022-06-21

**Authors:** Salimah Japar, Kensaku Fukunaga, Toshihiro Kobayashi, Hitomi Imachi, Seisuke Sato, Takanobu Saheki, Tomohiro Ibata, Takafumi Yoshimura, Kim Lam Soh, Swee Leong Ong, Zamri Muhamed, Koji Murao

**Affiliations:** 1 Department of Endocrinology and Metabolism, Faculty of Medicine, Kagawa University, 1750-1 Ikenobe, Miki-cho, Kita-gun, Kagawa 761-0793 Japan; 2 Department of Nursing, Faculty of Medicine and Health Science, Universiti Putra Malaysia, 43400 UPM Serdang, Selangor Malaysia; 3 School of Nursing Science, Faculty of Medicine, Universiti Sultan Zainal Abidin, Kampung Gong Badak, 21300 Terengganu, Malaysia; 4 Department of Medicine, Hospital Sultanah Nur Zahirah, Ministry of Health Malaysia, 20400 Kuala Terengganu, Malaysia

**Keywords:** Type 2 diabetes mellitus, d-allulose, Ramadan, postprandial glucose, CGM

## Abstract

**Background:**

During Ramadan fasting, postprandial hyperglycemia is commonly observed after *iftar* (break of fast at sunset) meal. d-allulose is a rare sugar and is reported to have several health benefits, including the suppression of increase in postprandial glucose levels. This study investigates whether d-allulose (a C-3 epimer of d-fructose) improves the postprandial glucose in patients with type 2 diabetes mellitus (T2DM) during Ramadan.

**Methods:**

This was a pilot, prospective single-arm study design that was conducted for 10 consecutive days; 5 days of control and 5 days of consumption. The primary outcome was postprandial peak glucose levels. During the consumption period, 8.5 g of d-allulose was consumed by the participants before *iftar* meal. Postprandial glucose was measured using a continuous glucose monitoring system.

**Results:**

A total of 12 participants completed the study. Significant lower (*p* < 0.01) postprandial glucose values and the glucose incremental area under the curve (iAUC) were observed from 0 to 180 min during the consumption period compared to the control period. The consumption period demonstrated significantly higher percentages of time in which glucose values were found in the target range (*p* = 0.0032), and when the glucose levels above the target range were reduced (*p* = 0.0015).

**Conclusions:**

The supplementation with d-allulose has the potential to improve postprandial hyperglycemia in patients with T2DM after *iftar* during Ramadan. Further studies are needed to confirm these findings.

*Trial registration* ClinicalTrials.gov NCT05071950. Retrospectively registered, 8 October 2021.

## Background

Reports from the International Diabetes Federation revealed that about 451 million people worldwide had diabetes in 2017, and the number is projected to increase to 693 million by 2045 [1]. Approximately 90 million Muslims worldwide have diabetes [2]. In Malaysia, the prevalence of diabetes is estimated as 18.3% affecting 3.9 million adults aged 18 years and above. The prevalence of diabetes increased rapidly from 13.4% in 2015 to 18.3% in 2019 [3]. Obesity due to higher intake of sweetened foods, carbohydrates, and sedentary lifestyles was revealed as the main factor of type 2 diabetes mellitus (T2DM) in Malaysia [4, 5].

Fasting during the month of Ramadan (the ninth month of the Islamic Calendar) is one of the five pillars of Islam. During Ramadan, all healthy Muslims are required to fast for a whole month from dawn to sunset. Fasting durations vary by geographical area [6], and in Malaysia, the duration is approximately 14 hours. Despite Ramadan fasting having several benefits [7, 8], fasting in individuals with uncontrolled diabetes increased the risk of hypoglycemia, hyperglycemia [9], diabetic ketoacidosis, and thrombosis due to dehydration [10, 11]. Although the holy Quran has explicitly exempted sick people from this obligation [12], many Muslim diabetic patients choose to fast despite the associated risks [10, 13, 14].

One of the underlining events leading to the onset of diabetes, especially in healthy individuals, is postprandial hyperglycemia. Postprandial hyperglycemia refers to a sudden increase in blood glucose levels above 140 mg/dL (7.8 mmol/L) within the first two hours after a meal [15]. It is a well-established risk factor for the onset and progression of vascular complications among diabetes patients. Postprandial hyperglycemia is usually observed during Ramadan, especially after *iftar* (break of fast at sunset) [16]. Despite the lower eating frequency during Ramadan, energy intake remains unpredictable and differs between individuals in line with dietary practices which are influenced by economic status, and dietary behaviors [16]. Ramadan is recognized as a month of feasting in several Muslim societies, including Malaysia [17]. A sudden rise in blood glucose levels occurs after *iftar* given that the meals are typically carbohydrate-rich, high-calorie, and usually sweet food. Additionally, most individuals consume more calories during *iftar* than *suhoor* (pre-dawn meal) and engage in less physical activities at night [16]. These events make it difficult for diabetes patients to effectively control and manage their blood glucose levels.

A potential solution to these issues may be d-allulose (a C-3 epimer of d-fructose) that is found in small quantities in nature. d-allulose is a monosaccharide, a rare sugar type with 70% the sweetness of sucrose and has zero calorie. Extensive basic and clinical studies [18–20] have reported beneficial outcomes from d-allulose for human health, including improved hypoglycemia, reduced postprandial hyperglycemia [19, 21] hypolipidemia, and antioxidant activities [21]. Based on the reports by the U.S. Food and Drug Administration (FDA), d-allulose is well-known as safe (GRAS) for use as a food ingredient and with other sweeteners [22]. Consumption of d-allulose led to significant glucose suppressive effects in healthy individuals after meals [18, 23, 24]. Similar findings were detected in pre-diabetes patients following the administration of d-allulose [19]. However, there is limited information and data paucity regarding the effects of d-allulose in diabetic patients. This study is the first attempt to elucidate the effects of d-allulose in response to normal routine meals consumed by patients with type 2 diabetes mellitus during Ramadan *iftar.* In this study, we hypothesized that supplementation with 8.5 g d-allulose prior to carbohydrate intake during *iftar* will reduce the postprandial blood glucose.

## Methods

### Study design

**Fig. 1:**
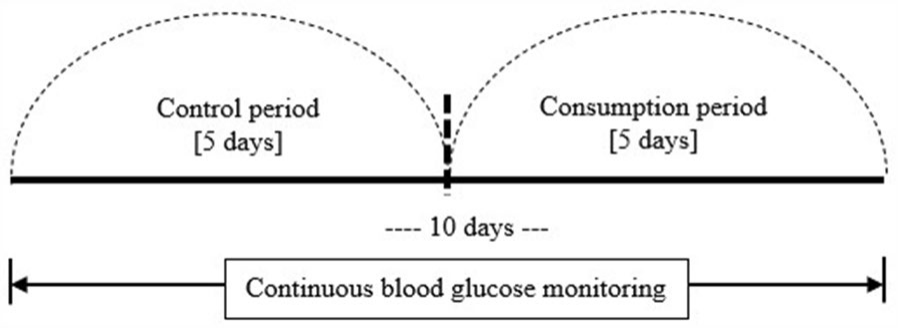
Graphic presentation of the study design. All participants underwent 10 days study period consecutively; began with 5 days control period and the following 5 days of consumption period

This research entailed a pilot, prospective intervention study involving a single-arm group. The study was carried out during Ramadan from April 13 to May 12, 2021. Specifically, the study duration was 10 consecutive days in the Month of Ramadan, comprising a control period and consumption period of 5 days each (Fig. 1).

The study protocol was registered on ClinicalTrials.gov (identifier: NCT05071950) and the National Medical Research Register (NMRR-19–3457-51288), whereas approval to conduct the study was obtained from the Medical Research Ethics Committee (MREC), Ministry of Health Malaysia (KKM/NIHSEC/P19-2734). Written informed consent was provided by the participants prior to enrollment and the study procedure were performed according to the principles of the Declaration of Helsinki 1964 and later versions.

### Participants

This study recruited Muslim T2DM patients aged 20–70 years old. The inclusion criteria were: patients with HbA1c levels of < 8% before Ramadan, treated with oral hypoglycemic agents (OHAs) and/or diet control, intending to fast for Ramadan, and categorized in the low-risk group [25]. Patients were excluded if they had serious complications of diabetes, serum creatinine level of 1.5 mg/dl or higher, pregnant, breastfeeding, or who were advised not to fast by a doctor. This study was conducted at the diabetes outpatient clinic of a tertiary government hospital located in East Peninsular Malaysia. The G*Power 3.1.9.4 software was used to estimate the sample size of the study [26]. Using a statistical power of 95%, significance level of 0.05, and effect size of 1, based on previous study [18] it was estimated that at least 40 participants (20% of expected attrition rate was added) would be needed to show a statistically significant difference in glycemic control between 2 weeks of Ramadan fasting.

### Data collection

The screening and enrollment processes were conducted within 2 months before Ramadan. This study required participants to visit the research site twice. Vital and physical parameters such as heart rate, blood pressure, height, weight, and waist circumference were measured on the first visit. Participants received seven packs of 8.5 g d-allulose and a food diary along with oral and written instructions. A sensor of flash continuous glucose monitoring (FCGM) device was attached to each participant. On the final visit, the sensors were removed and data were downloaded using a reader. Thereafter, the food diaries were collected and the side effects were evaluated. Hypoglycemia and hyperglycemia symptoms were recorded based on participants’ self-reports.

Clear zipped packs were used to collect 8.5 g of d-allulose with over 99% purity (Matsutani Chemical Industry Co., Ltd.). Each pack was numbered (1 to 7), indicating the corresponding days and dates. During the consumption period, participants dissolved one pack of d-allulose in plain water and finish the drink before consuming their main *iftar* meal. The quantity of plain water was based on participants’ preferences. During the study, no additional sugar/sweetener drinks were allowed before the *iftar* meals. Participants recorded the type and amount of *iftar* meals and drinks they consumed, and intakes of any additional sugar after the *iftar* in the food diary. During both periods, participants were instructed to adhere to the standard diabetes management provided by their health professionals, as there were adjustments in timing and doses of OHAs, as well as caloric intakes for Ramadan [25, 27]. No new or modified drugs were added or changed during the period. Therefore, no changes in diabetes treatment were recorded either before or when consuming d-allulose.

### Glycemic measures

The FreeStyle Libre Pro Flash Glucose Monitory system (Abbott Japan Diabetes Care Inc., Tokyo, Japan) was used to measure the glucose values. This continuous glucose monitoring device measures glucose in the interstitial fluid. This device is reliable, non-invasive, and consists of a reader and a small sensor. The range target glucose values were set between 70 and 140 mg/dl (3.9–7.8 mmol/L). The FCGM sensor recorded interstitial glucose levels in 15-min intervals for a total of 96 readings every 24 h. However, only 10 days (5 days each of the control and consumption period) of data at 0 min (before i*ftar*/ d-allulose consumption) and 15, 30, 45, 60, 75, 90, 105, 120, 135, 150, 165 and 180 min after *iftar*/ d-allulose consumption were extracted for data analysis. Data from the first two days and the last two days were excluded as they were considered unstable [28]. Participants took approximately 15–30 min to complete their meal during *iftar*. Therefore, data at the beginning of *iftar* were included in the analysis.

### Outcome measures

The primary outcome was the peak postprandial glucose level documented by the FCGM. Secondary outcomes were: (1) percentage of time in which postprandial glucose levels were in the target range (%TIR = 70–140 mg/dl), (2) percentage of time in which glucose levels were above the target range (%TAR =  > 140 mg/dl), (3) the percentage of time in which glucose levels were below the target range (%TBR =  < 70 mg/dl), (4) side-effects of d-allulose, and (5) hypoglycemia or hyperglycemia symptoms reported by the participants.

### Statistical analyses

The trapezoidal method was employed to calculate the glucose increment area under the curve (iAUC). The average glucose TIR, TAR, and TBR percentages were analyzed within 180 min after *iftar.* Meanwhile, all statistical analyses were undertaken using SPSS version 25 (IBM Corp, New York, USA) software. Results were presented in graphs developed using Microsoft Excel. Frequencies and percentages were used to express the categorical variables, whereas normally distributed data (continuous variables) were summarized using means and standard deviations (SD). Glucose levels between the control and consumption periods and differences within repeated days were assessed using the one-way repeated measure ANOVA with Bonferroni post hoc test. The paired sample *t*-test was used to compare the average glucose TIR, TAR, and TBR percentages. A p-value less than 0.05 was considered for statistical significance in all the tests conducted.

## Results

### Recruitment and participant characteristics

**Fig. 2:**
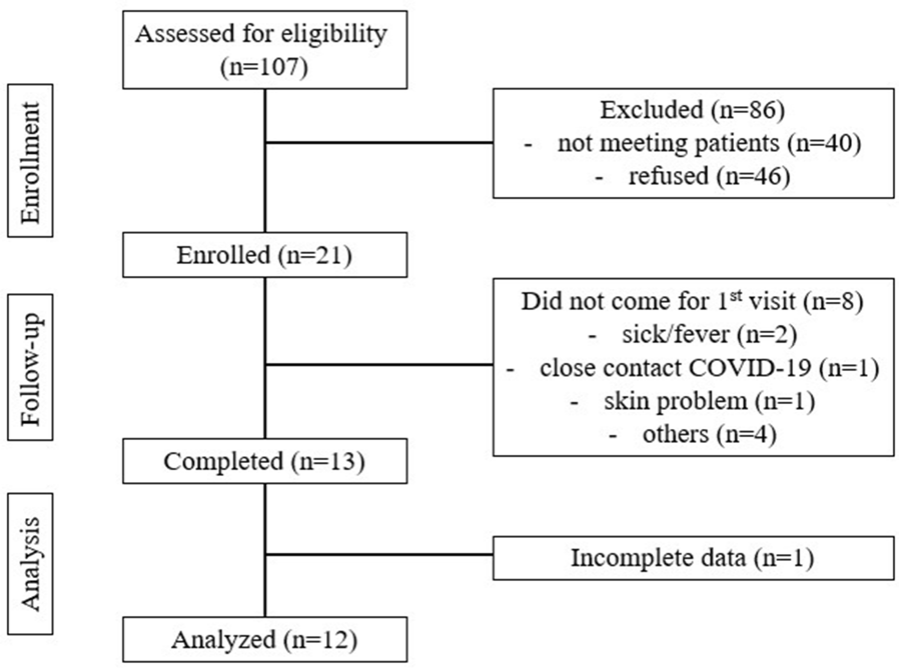
The flow chart of participant enrollment

A total of 107 T2DM patients who were eligible were recruited in this study. Of these, 21 provided their consent but only 12 participants completed the final analysis (Fig. 2). Overall, equal numbers of males and females were obtained in this study, with a mean age of 55.2 ± 6.83 years. Meanwhile, the participants’ HbA1c pre-Ramadan, duration of diabetes, and BMI were 6.7 ± 0.41%, 6.6 ± 6.3 years, and 32.2 ± 7.6 kg/m^2^, respectively. More than half (66.7%) of the participants received a combination of two OHAs for diabetes treatment (Table 1).

**Table 1:** Characteristics of study participants

Variables	Mean ± SD	n (%)
Gender (Male/Female**)**		
Male		6 (50)
Female		6 (50)
Age (years old)	55.2 ± 6.83	
Employment		
Self-employment		3 (25.0)
Government		7 (58.3)
Unemployment		1(8.3)
Retired		1 (8.3)
Education level		
High school		7 (58.3)
University/College		5 (41.7)
Duration of T2DM (year)	6.6 ± 6.3	
HbA1c (%)	6.7 ± 0.41	
Height (cm)	160 ± 9.4	
Weight (kg)	82.7 ± 19.8	
BMI (kg/m^2^)	32.2 ± 7.6	
WC (cm)	97.7 ± 10.9	
SBP (mmHg)	138 ± 14.9	
DBP (mmHg)	88 ± 10.2	
Pulse (beats/min)	78 ± 10.4	
OHAs		
Metformin		3(25)
Metformin & Sulfonylurea		8 (66.7)
Diet control		1 (8.3)

*HbA1c* glycated haemoglobin, *BMI* body mass index, *WC* Waist circumference, *SBP* systolic blood pressure, *DBP* diastolic blood pressure, *OHAs* oral hypoglycemic agents

### Effect of d-allulose on postprandial peak glucose

**Fig. 3:**
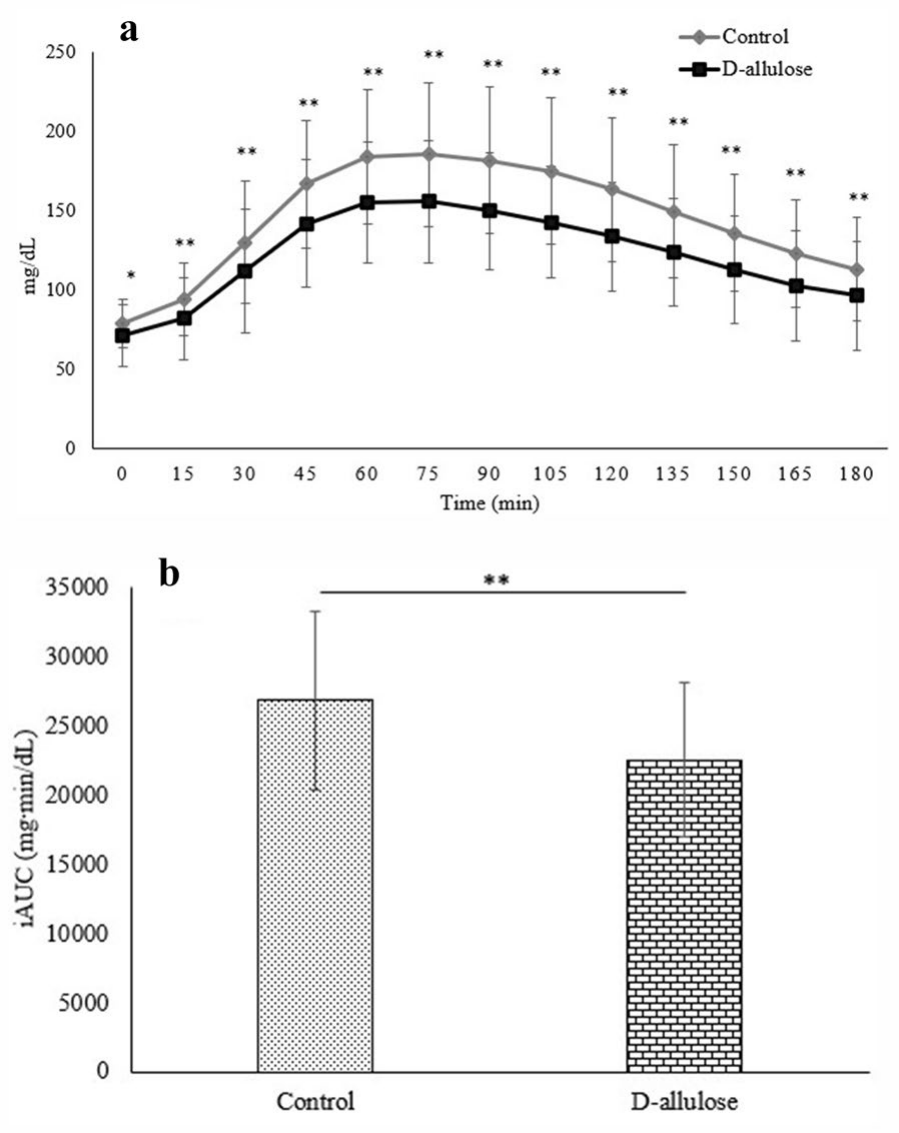
The comparison of postprandial glucose at control and consumption (D-allulose) periods. Figure 3a shows postprandial glucose at 0 (pre) to 180 min after *iftar* and Fig. 3b shows the average glucose incremental area under the curve (iAUC) (3b) within 180 min after *iftar.* Data reported as mean ± SD of glucose values of 5 days of the control period and 5 days of the consumption period. Postprandial glucose levels were significantly different between both periods (*p < 0.05; **p < 0.01)

Figure 3a and b show the mean postprandial glucose values and the glucose iAUC after *iftar* at 0–180 min, respectively. The participants’ postprandial glucose values were significantly lower at each time point (after *iftar* at 0–180) in the consumption period compared to the control period (*p* < 0.01) (Fig. 3a). Likewise, the postprandial glucose iAUC was significantly attenuated during the consumption period (22,474.1 ± 5576.8 mg∙min/dl) compared to the control period (26,792.3 ± 6411.9 mg∙min/dl; *p* < 0 0.01) (Fig. 3b).

### Percentage of time: glucose in range

**Fig. 4:**
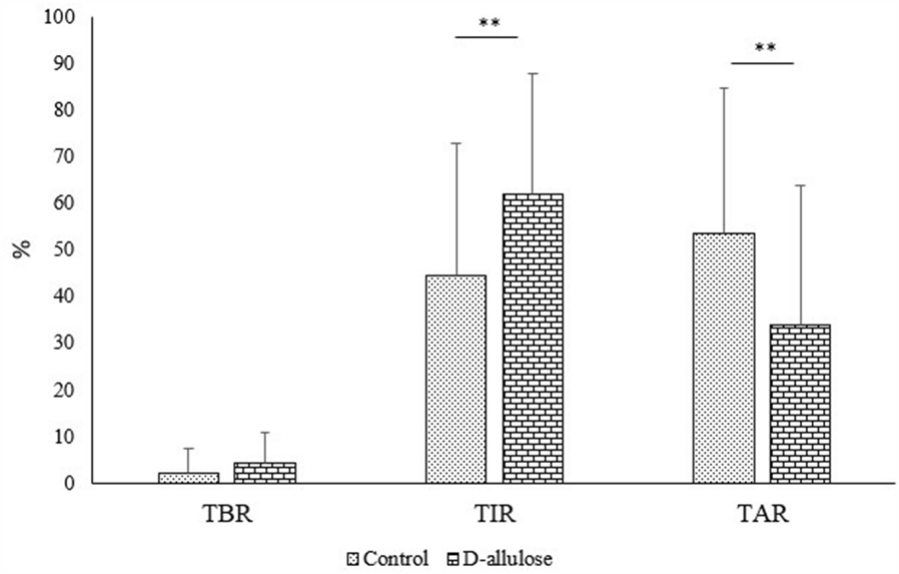
The effect of D-allulose during the consumption period compared to control period on the percentage of time postprandial glucose in-target range (%TIR), percentage of time glucose above-target range (%TAR) and percentage of time glucose below-target range (%TBR). Percentages of TIR, TAR and TBR were analyzed by obtaining the average of frequency glucose in TIR, TAR and TBR for 5 days (control and consumption periods) at 15 to 180 min after *iftar*. The average then divided by 12 times point and multiple with 100. Data reported as mean ± SD. There were significant differences of postprandial glucose between both periods (**p < 0.01)

**Fig. 5:**
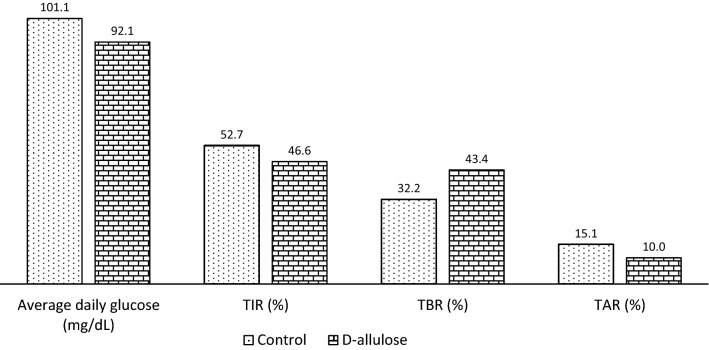
The average 24-h glucose changes, average % of TIR, TBR and TAR for 5 days (control and consumption periods). Data reported as mean and percentage (%)

Figure 4 shows the comparison of %TIR, %TBR, and %TAR within 180 min after iftar between the control and consumption periods. During the consumption period, the %TIR was significantly higher (62 ± 25.99% vs. 44 ± 28.48%; *p* = 0.0032) and %TAR was significantly lower (34 ± 29.84% vs. 54 ± 31.08%; *p* = 0.0015) than in the control period. No significant difference was observed in the %TBR. Figure 5 shows the 24-h glucose changes throughout the study period. The average daily glucose was lower in the consumption period than in the control period, with 92.1 mg/dL and 101.1 mg/dL, respectively. The lower values in the consumption period were also reported for %TIR and %TAR. No significant difference in glucose changes was observed between the control and consumption periods.

### Side-effects of d-allulose

Regarding the side effects of d-allulose, four events of minimally loose stool were reported among two participants. One participant experienced one instance in the early morning after consuming d-allulose on days 3, 4, and 5, whereas the other participant reported a similar event at night on the last day of administration. Nevertheless, no other side effects were observed. Symptomatic hypoglycemia and hyperglycemia were also not reported by participants.

## Discussion

This pilot study was conducted during Ramadan when Muslims fast in the daytime from sunrise to sunset. A small number of participants completed this study. The participants consumed 8.5 g of d-allulose before the *iftar* meal for five consecutive days. Resultantly, the postprandial glucose values and the iAUC were significantly lower during the d-allulose period. The %TIR also increased while the %TAR decreased in comparison to the control period. Hence, we accepted our hypothesis.

Previous studies have shown that supplementation of d-allulose with carbohydrate intake improved postprandial glucose metabolism [29, 30]. Clinical studies in pre-diabetes and T2DM patients revealed a significant reduction in postprandial glucose levels, glucose iAUCs, and insulin levels following d-allulose supplementation [19, 31]. Furthermore, compared to healthy individuals, administration of d-allulose led to significantly lower blood glucose levels among pre-diabetes [19] and diabetes patients [31].

In the present study, 8.5 g of d-allulose was effective in suppressing glucose elevation before carbohydrate intake in diabetic patients in Malaysia. The amount of d-allulose used in this study was calculated based on the average carbohydrate intake of Malaysians [19, 32], and it has not been tested in previous studies. Prior clinical research reported that a minimum of 5 g to 10 g of d-allulose before carbohydrate loading is sufficient to reduce postprandial blood glucose levels, insulin levels, and glucose iAUCs among pre-diabetes individuals [19] and diabetic patients [31]. Nonetheless, the outcomes in healthy subjects may vary. Postprandial blood insulin and glucose levels were significantly suppressed in Japanese subjects administered 5 g and 7.5 g of d-allulose on 75 g maltodextrin [18] and 5 g of d-allulose in response to standardized meals [19, 24]. However, a recent study in the United States showed insulin levels were only significantly reduced with 10 g of d-allulose [23]. A Canadian study failed to demonstrate a suppressive effect of d-allulose [33]. The researchers suggested that the wide inter-individual variation in participants’ glucose responses might explain the results. Additionally, no effect was detected in blood glucose and insulin levels following a single administration of 7.5 g d-allulose in healthy participants, thus reflecting that hypoglycemia is not induced by d-allulose [18]. Overall, d-allulose showed better suppression of postprandial glucose elevation among people with impaired glucose tolerance but studies on diabetes patients are still limited.

Insulin levels were not measured in this study due to the limitations in the function of the FCGM devices. This is the first attempt to investigate participants’ real-carbohydrate intake. Nevertheless, the participants’ carbohydrate and calorie intake were not analyzed but they were instructed to record the quantities of their *iftar* meals in their food diaries as supporting information. Overall, the menu differed among the participants but the nutritional intake or changes in the menu were not significantly different in each participant during control and d-allulose periods. Most of them consumed 150 g of rice on average apart from other side dishes and desserts. In Muslim societies, including Malaysia, it has become a tradition where *iftar* is celebrated as a mini feast to mark the break of fast on that day with family members or friends. In general, Malaysian dietary patterns contain high sugar and fat intakes, and compared to other ethnic groups, Malays consume high-calorie meals during daily intake [34]. Hence, maintaining a healthy diet during Ramadan is a challenge for individuals with diabetes [35].

The possible underlying mechanisms explaining the role of d-allulose in suppressing postprandial glucose levels and enhanced insulin regulation following the consumption of carbohydrate foods have been demonstrated in previous animal studies. One of the underpinning events is the inhibition of carbohydrate-digesting enzymes [36–38] or competition with glucose transporters in small intestine [39, 40], thus reducing intestinal glucose absorption. Findings suggested that d-allulose shares the same sugar transporters of d-glucose and d-fructose in the small intestine [39, 40]. Competition for the transportation of d-glucose and d-fructose was elicited due to the presence of d-allulose, thereby reducing the permeation rate of both sugars These were evidenced by an in vitro study conducted in Caco-2 monolayer cell lines. The glucose permeability was reduced by 60% when the addition of 30 mM allulose to 30 mM glucose was combined [40]. Another study on rats showed Tracer d-[14C]-fructose uptake was reduced to 54.8% in 50 mM d-allulose [39]. Furthermore, rats’ studies revealed that d-allulose stimulated glucagon-like peptide-1 secretion and enhanced insulin release, as a result improved glucose tolerance [41, 42] and provided a therapeutic effect on pancreatic β-cell function [37]. Immunohistochemical analyses demonstrated healthy pancreatic β-cell were clearly observed in both T2DM rat models and normal rats treated with d-allulose over 60 weeks; meanwhile, hyperplastic changes and extensive fibrosis were seen in control T2DM rat models [37]. These findings suggest supplemental d-allulose has potential effects on diabetes prevention and treatment.

This is the first study in which FCGM was used to measure glucose levels following d-allulose supplementation. Previous studies had primarily used antecubital veins or fingertips for blood glucose measurements. Within 180 min after *iftar*, the %TAR decreased and %TIR increased significantly compared to the control period. This indicated that d-allulose shortened the hyperglycemic phase and maintained the glucose level in the normal target range for a longer time compared to the control period. A critical phase occurs after a meal in which glucose is expected to reach the target within two hours of food ingestion to improve postprandial hyperglycemia, thus preventing the progression of microvascular and macrovascular complications in T2DM patients [15]. Nevertheless, glycaemic targets may differ depending on the severity of diabetes.

Two participants reported an instance of a minimally loose stool after d-allulose consumption, and these were not considered diarrhea. Studies have shown that d-allulose is safe for consumption in healthy individuals [19, 43] and T2DM patients [44]. A gastrointestinal tolerance test on healthy participants [43] reported that severe diarrhea was noted only at a dose of 0.5 g/kg of body weight. Two long-term safety evaluation studies reported no significant clinical concerns after taking 5 g of d-allulose thrice daily [19, 44]. The present pilot study had some limitations. Given that the study was conducted during the COVID-19 pandemic under the restricted movement order implementation by the Malaysian government, the outpatient clinic had to adhere to strict standard operating procedures. As a result, the number of appointments in a given period was reduced and only a few patients could participate in this study. These limitations contributed to the lack of a high-quality study design for more precise outcomes in this research. The FCGM is relatively new in Malaysia and its use during Ramadan was frowned upon by some patients who wished to have Ramadan free of intrusions due to religious and cultural commitments. Furthermore, this study only involved Malay patients at a single medical institution; thus, the findings cannot be generalized to other patients at other medical institutions. The outcomes could be better clarified in future studies by enrolling a larger sample size.

## Conclusions

Our findings suggest that supplementation with 8.5 g of d-allulose before *iftar* meal revealed a significant suppressive effect on postprandial glucose elevation. This study also confirmed that no severe side effects of d-allulose or symptomatic hypoglycemia or hyperglycemia were reported throughout the study period. We believe d-allulose has its potential role in improving glycemic control in T2DM patients, however it cannot be applied clinically immediately. These findings will support the need for future clinical studies with adequate power and considering other parameters that might affect glycemic changes during Ramadan fasting.

## Data Availability

The datasets used and/or analysed during the current study are available from the corresponding author on reasonable request.

## Abbreviations

ANOVA: Analysis of variance
BMI: Body mass index
DBP: Diastolic blood pressure
HbA1c: Glycated haemoglobin
iAUC: Increment area under the curve
SBP: Systolic blood pressure
TAR: Above-target range
TBR: Below-target range
TIR: In-target range
T2DM: Type 2 diabetes mellitus.
WC: Waist circumference
OHAs: Oral hypoglycemic agents
